# Myeloid Metabolism as a New Target for Rejuvenation?—Comments on Restoring Metabolism of Myeloid Cells Reverses Cognitive Decline in Ageing. Nature. 2021 Feb;590(7844):122-128

**DOI:** 10.20900/immunometab20210034

**Published:** 2021-10-31

**Authors:** Marlen Knobloch, Rosa C. Paolicelli

**Affiliations:** Department of Biomedical Sciences, University of Lausanne, Lausanne 1005, Switzerland

**Keywords:** myeloid cells, ageing, metabolism, rejuvenation, prostaglandin E_2_

## Abstract

Research led by Katrin Andreasson suggests that fixing age-induced metabolic defects in myeloid cells would suffice to reverse cognitive impairment and to restore synaptic plasticity to the level of young subjects, at least in mice. This opens up the possibility to develop rejuvenating strategies by targeting immune dysfunction.

Ageing is accompanied by many molecular and cellular alterations, which lead to a gradual deterioration of physiological functions. While lifestyle and genetics have a big impact on the speed of these ageing processes, ageing per se is usually seen as not easy to revert [1]. Neurocognitive changes are well established features associated with ageing. While cognitive decline is clearly underlined by synaptic and neuronal dysfunction, other players are emerging as critical elements in the equation of brain ageing. Inflammation seems to be one of those, with pro-inflammatory factors being associated with poor cognitive performance [2,3], pointing to immune cells as important regulators in this process.

The immune system is drastically affected with ageing [4]. While the adaptive immune response comprising B- and T-Cells is diminished, the innate immune system (i.e., cells of the myeloid lineage) shows an increase in the pro-inflammatory state, also known as “inflammaging” [5,6]. This chronic low-inflammatory state is mainly driven by macrophages and pro-inflammatory cytokines.

Cellular metabolism has emerged as a key player in the regulation of immune function [7–9], starting already at the level of myeloid versus lymphoid lineage decision [10,11] and greatly affecting cellular behaviour in the mature immune cells. Several recent studies have suggested that an altered cellular metabolism in aged macrophages might directly contribute to the pro-inflammatory signature [12–14]. However, the detailed mechanisms initiating this increased inflammation with aging remain unclear.

In a recent publication, Minhas and colleagues have elucidated this cascade using an impressive set of in vitro and in vivo experiments in mice and in human myeloid cells [15]. They found that aged myeloid cells have a decrease in cellular respiration, measured by oxygen consumption rate (OCR), and a decrease in glycolysis, measured by the extracellular acidification rate (ECAR), suggesting that aged myeloid cells undergo a general bioenergetic failure. The proposed driving cause is the increased prostaglandin E_2_ (PGE_2_) signaling in the ageing myeloid compartment, mediated by the age-dependent upregulation of EP_2_, one of the four PGE_2_ receptors. They show that this signaling cascade is also altered in human monocyte-derived macrophages (MDMs). PGE_2_, also known as dinoprostone, is a lipid messenger which acts as a key mediator of inflammation, downstream of the cyclooxygenase 2 (COX-2) pathway. Among other responses, this increase leads to the activation of glycogen synthase (GYS1), the key enzyme converting glucose into glycogen. This channeling of glucose away from glycolysis might thus be at the origin of the metabolic dysfunction of macrophages.

Conditional knockout of EP_2_, specifically in the myeloid cells (Cd11b-CRE;EP_2_ cKO) of aged mice proves to be indeed an effective strategy at multiple levels. First, it rescues the expression of some of the immune factors upregulated with age, both in the plasma and in the hippocampus. Second, the loss of EP_2_ also reduces glycogen levels, normalizing the metabolic state and the associated mitochondrial defects observed in old macrophages. A similar effect is also mimicked by directly inhibiting GYS1 via shRNAs in human MDMs. Third, strikingly, aged EP_2_ cKO mice appear to be completely protected from a decline in hippocampal-related memory functions with ageing.

Interestingly, the same group showed that these mice displayed beneficial effects when crossed to a mouse model of Alzheimer’s disease. EP_2_ cKO microglia were able not only to promote Aβ clearance, but also to prevent synaptic injury and memory deficits [16]. However, the Cd11b-CRE line affects the whole myeloid lineage, including monocytes, macrophages, and microglia. Thus, the specific contribution of each of those cell populations in mediating the positive outcome of EP_2_ ablation remained somewhat unclear. With their recent study, Minhas et al. tried to disentangle this further [15]. By injecting a brain-impermeant EP_2_ antagonist in aged mice, they could show a significant reduction in the levels of age-associated immune factors and a global restoration of the metabolic profile. This treatment is sufficient to rescue the age-related long term potentiation defects in the hippocampus, along with cognitive function.

Overall, these data support an upstream role of peripheral myeloid cells in orchestrating the process of brain ageing, underscoring the important cross-talk between the immune and the central nervous systems. This study nicely illustrates the importance of the cellular metabolic state of myeloid cells: it highlights that not only the availability of glucose, but also its channeling into different pathways (glycolysis versus glycogen synthesis) contributes to maintaining proper myeloid function (Figure 1).

Surprisingly, restoring the PGE_2_ signaling in myeloid cells to a youthful state is enough to prevent age-dependent cognitive decline However, it should be noted that, although GYS1 knockdown recovered aging-related metabolic and inflammatory changes in human macrophages, all interventions reversing molecular and cognitive changes in aged mice were performed by inhibiting the whole signaling downstream to E_2_ receptors. Thus, the improved cognitive outcome could also be the result of the general attenuation of PGE_2_ signaling, not only due to restored metabolic function. Furthermore, it was recently shown that that aging-related sarcopenia is mediated by decreased PGE_2_ signaling in both myofibers and muscle-resident macrophages [17]. Strikingly, restoration of PGE_2_ signaling prevented sarcopenia in old mice, while its disruption was sufficient to induce sarcopenia in young mice [17]. These data suggest that PGE_2_-mediated effects during aging might be tissue-specific and these differences should be considered in future therapeutic approaches.

## Figures and Tables

**Figure 1:**
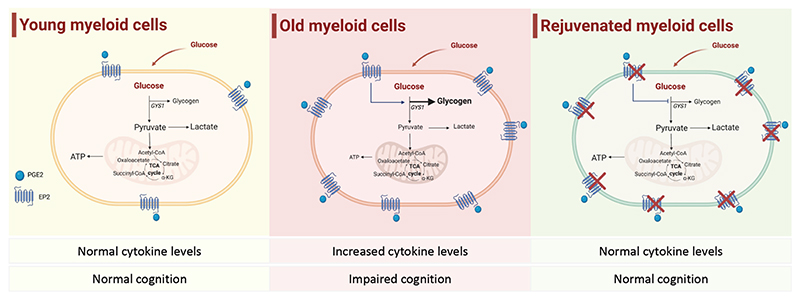
Old myeloid cells (**center**), as opposed to young ones (**left**), exhibit increased PGE_2_-EP_2_ signaling, associated with increased glycogen levels, suppression of glycolytic flux and compromised mitochondrial function. Genetic or pharmacological inhibition of the PGE_2_-EP_2_ signaling, or of the glycogen synthase (GSY1) activity is sufficient to ‘rejuvenate’ myeloid cells (**right**), normalizing the levels of glycogen, and restoring both glycolytic flux and mitochondrial oxidative phosphorylation. Figure created with BioRender.com.
